# Pharmacological management of hypertension and outcome among patients on hemodialysis at Muhimbili National Hospital, Tanzania: a cross-sectional study

**DOI:** 10.11604/pamj.2023.46.67.39778

**Published:** 2023-10-24

**Authors:** Devis Mhagama, Manase Kilonzi, Peter Kunambi, Deus Buma, Fredrick Kalokola, Paschal Ruggajo, Ritah Francis Mutagonda

**Affiliations:** 1 Dodoma Christian Medical Center Trust, Dodoma, Tanzania,; 2 Department of Clinical Pharmacy and Pharmacology, School of Pharmacy, Muhimbili University of Health and Allied Sciences, Dar es Salaam, Tanzania,; 3 Department of Clinical Pharmacology, School of Biomedical Sciences, College of Medicine, Muhimbili University of Health and Allied Sciences, Dar es Salaam, Tanzania,; 4 Department of Pharmacy, Muhimbili National Hospital, Dar es Salaam, Tanzania,; 5 Department of Internal Medicine, Weill Bugando School of Medicine, Mwanza, Tanzania,; 6 Directorate of Curative Services, Ministry of Health, Dodoma, Tanzania

**Keywords:** Hypertension, hemodialysis, antihypertensive medications, blood pressure, Tanzania

## Abstract

**Introduction:**

hypertension is prevalent among patients attending hemodialysis. However, published information on hypertension management among patients on hemodialysis in African countries is scarce. This study assessed antihypertensive medication prescribing patterns and blood pressure control among patients with hypertension on hemodialysis in Tanzania.

**Methods:**

an analytical cross-sectional study was conducted at Muhimbili National Hospital in Dar es Salaam from April to June 2022. The study population consisted of patients with hypertension undergoing hemodialysis. Data on demographic, clinical characteristics and the antihypertensive medications used by the patients was collected using a structured questionnaire. Analysis was performed using Statistical Package for the Social Sciences software version 26. Uncontrolled pre-dialysis blood pressure determinants were assessed using a modified Poisson regression model. A p-value < 0.05 was considered statistically significant.

**Results:**

out of 314 participants, the majority (68.2%, n= 214) were male, and the median age was 52 (interquartile range: 42, 60) years. Only 16.9% (n= 53) of patients had their pre-dialysis blood pressure controlled. The most frequent antihypertensive medications prescribed were calcium channel blockers (73.2%, n= 230). Patients with less than three dialysis sessions were 20% more likely to have uncontrolled blood pressure than those with three sessions in a week (adjusted prevalence ratio = 1.2).

**Conclusion:**

most patients on hemodialysis with hypertension had poor blood pressure control, according to the study. Patients with hypertension should be strongly encouraged to adhere to at least three hemodialysis treatments to achieve optimal blood pressure control.

## Introduction

Patients with severe and irreversible chronic renal disease or end-stage renal disease (ESRD) require routine dialysis. Dialysis patients frequently have hypertension because the kidneys are less efficient in maintaining blood pressure (BP) homeostasis [1]. In patients with ESRD getting renal replacement therapy with hemodialysis (HD), hypertension is a relatively common condition ranging from 72% to 88% of patients. It is often ineffectively treated, with only 30% to 50% reporting having well-controlled BP [2-5]. Poor BP management is a well-established risk factor for cardiovascular diseases (CVDs) and shorter survival in HD patients [6]. Volume overload and sodium excess are the primary factors exacerbating hypertension in HD patients [7]. To maintain hypertensive HD patients' BP at target levels (i.e. pre-dialysis BP 140/90 mmHg and post-dialysis BP 130/80 mmHg), non-pharmacological management techniques like dietary sodium restriction, gradual dry weight loss, and customized dialysate sodium prescriptions are advised as initial strategies [8]. However, it is recommended to consider antihypertensive drugs for HD patients who do not achieve adequate BP control using non-pharmacological approaches [8]. According to previously published studies, antihypertensive drugs have been positively associated with lower cardiovascular morbidity and mortality in HD patients [9,10]. Based on a recent consensus, all major antihypertensive classes effectively treat hypertension in HD patients except for diuretics [8]. Diuretics are not recommended for treating hypertension in HD patients due to the potential for ototoxicity in anuric persons and the minimal alteration in central hemodynamic parameters [8,11-13]. Beta-blockers (BBs) are preferable for treating hypertension in HD patients because of their cardioprotective effects [14,15]. Using Angiotensin-Converting Enzyme Inhibitors (ACEIs) is linked to improved survival, according to the findings of the Efrati *et al*. 2002 study [16]. Combining ACEIs and Angiotensin receptor blockers (ARBs) can enhance BP control and cardiovascular outcomes [8]. Nevertheless, patients with volume overload frequently take calcium channel blockers (CCBs) and other vasodilators because they efficiently control BP [17]. According to studies, amlodipine could drop systolic BP by about 10mmHg less than placebo without causing intradialytic hypotension [18]. The guidelines recommend managing HD patients using a combination of antihypertensive agents to achieve adequate BP control [13]. Despite the reported high proportion (96%) of hypertension in HD patients globally, including sub-Saharan African countries [19], there is scarce information on which antihypertensive medications have a better outcome and the overall BP control among this population in an African context. Therefore, this study assessed antihypertensive medications prescribing patterns and BP control among patients with hypertension on HD in Tanzania.

## Methods

**Study design, duration, and setting:** this was a hospital-based cross-sectional study conducted between April and June 2022. The study was conducted at Muhimbili National Hospital (MNH) at Upanga and Mloganzila branches. Muhimbili National Hospital is a public national referral and teaching hospital in Tanzania attending more than >25% of HD patients (approximately 385 patients per month) all over Tanzania with more than 42 HD machines [20]. Additionally, the hospital has qualified staff and modern facilities for managing patients with hypertension receiving HD.

**Study population:** the study included all hypertensive adult patients (18 years or above) on maintenance HD therapy for at least three months at MNH dialysis centers. Patients with mental disorders, bedridden patients, and pregnant women were excluded.

**Sample size and sampling strategy:** using the cross-sectional study formula, the sample size was estimated based on the prevalence of 27.8% controlled BP reported by Ahmad *et al*., 2020 among HD patients in Pakistan [21]. Therefore, the minimum sample size was 314 study participants. A consecutive sampling technique was used to recruit participants in the study.

**Data collection:** using a structured questionnaire, the principal investigator and research assistants collected data on the sociodemographic characteristics of the patients, including their age, sex, level of education, employment status, use of tobacco products, and alcohol consumption. Clinical and laboratory data such as body mass index (BMI), anthropometric measurements, vascular access, hemoglobin level, and blood urea nitrogen were also recorded. Information on comorbidities and medication use were collected by reviewing patient files and prescriptions to reduce recall bias. Patients with heart failure, left ventricular hypertrophy, coronary artery disease, or any other chronic cardiac disease were categorized as having CVDs. Antihypertensive medications prescribed to patients were categorized under their respective pharmacological classes. Study participants on a single antihypertensive were categorized as being on monotherapy, and those on more than one antihypertensive medication (either two different single drugs or a fixed-dose combination) were defined as being on multitherapy. The BP was measured and recorded by trained dialysis nurses using an oscillometer BP monitor. The average of two consecutive readings taken at 5-minute intervals of quiet rest while the patient was seated was used to determine the pre- and post-dialysis BP readings on the day of the hemodialysis session.

**Data analysis:** data was analyzed using IBM Statistical Package for the Social Sciences (SPSS) for Windows, Version 26.0 (IBM Corp. Released 2019. Armonk, NY). Frequency and percentages were used to summarize categorical variables, while the median and interquartile range (IR) was used for continuous variables. The Wilcoxon signed ranks test was used to measure the change in median BP before and after dialysis. Uncontrolled hypertension was diagnosed when pre-dialysis BP was ≥140/90 mmHg. A modified (robust) Poisson regression model was used to determine predictors of uncontrolled pre-dialysis BP among patients. Only variables with a univariate p-value < 0.2 were included in the multivariable regression model to be able to obtain adjusted prevalence ratio (aPR), and a p-value of < 0.05 was considered statistically significant.

**Ethical considerations:** the study was done after obtaining authority from Muhimbili University of Health and Allied Sciences (MUHAS) Institutional Review Board with reference number DA.282/298/01C/1085. Muhimbili National Hospital´s administration gave permission to enroll patients at the facility during the study period and a written informed consent was provided by the patients prior to being enrolled.

## Results

**Sociodemographic and clinical characteristics of the study participants:** a total of 314 participants were included in the final analysis, with a median age of 52 years. Of all participants, 68.2% (n= 214) were male, and 66.9% (n= 210) had health insurance schemes. The participants' median BMI was 22.5kg/m^2^, and the median hemoglobin level was 9.0g/dL. Moreover, 77.7% (n= 244) of participants had been hypertensive for over 12 months. Most (66.6%, n = 209) patients attended three sessions weekly. The characteristics are further described in Table 1.

**Table 1:** socio-demographic and clinical characteristics of the participants (n= 314)

Frequency (n)	Percent (%)	P-value
**Age group (years)**		
18 – 40	76	24.2
41 – 60	161	51.3
>60	77	24.5
**The median age in years (IQR)**		
52 (42- 60)		
**Sex**		
Male	214	68.2
Female	100	31.8
**Educational status**		
Informal education	9	2.9
Primary education	21	6.7
Secondary education	237	75.5
Tertiary education	47	15
**Insurance status**		
Fully insured	210	66.9
Not insured	104	33.1
**Alcohol use**		
Current drinker	5	1.6
Ex-drinker	48	15.3
Never-drinker	261	83.1
**History of other co morbidities**		
Yes	137	43.6
No	177	56.4
**Months since dialysis initiation**		
≤ 12	147	46.8
13 - 60	145	46.2
61 - 120	20	6.4
>120	2	0.6
The median (IQR) duration on dialysis is 16 (6-36) months		
**Dialysis sessions in a week**		
<3	105	33.4
3	209	66.6
The median (IQR) dialysis sessions is 3 (2-3) per week		
**Months since the hypertension diagnosis**		
≤ 12	70	22.3
13 - 60	140	44.6
60 - 120	70	22.3
>120	34	10.8

The median (IQR) duration to diagnose hypertension is 48 (19-72) months

**Antihypertensive medications prescribing pattern:** the most prescribed antihypertensive classes, either alone or in combination, were CCBs (73.2%, n= 230), followed by vasodilators (55.1%, n= 173), BBs (36.3%, n= 114), diuretics (24.9%, n= 78), ARBs (4.8%, n= 15), and ACEIs (1.3%, n= 4) (Table 2).

**Table 2:** antihypertensive medications prescribing pattern (n= 314)

Medication class	Frequency(n)	Percent (%)
BBs	114	36.3
CCBs	230	73.2
Diuretics	78	24.9
Vasodilators	173	55.1
ACEIs	4	1.3
ARB	15	4.8
Other antihypertensives	12	3.8
Number of antihypertensive prescribed 1	88	28.0
2	121	38.5
≥3	105	33.5

BBs: beta-blockers; ACEIs: angiotensin-converting enzyme inhibitors; CCBs: calcium channel blockers; ARB: angiotensin receptor blockers

**Figure 1:**
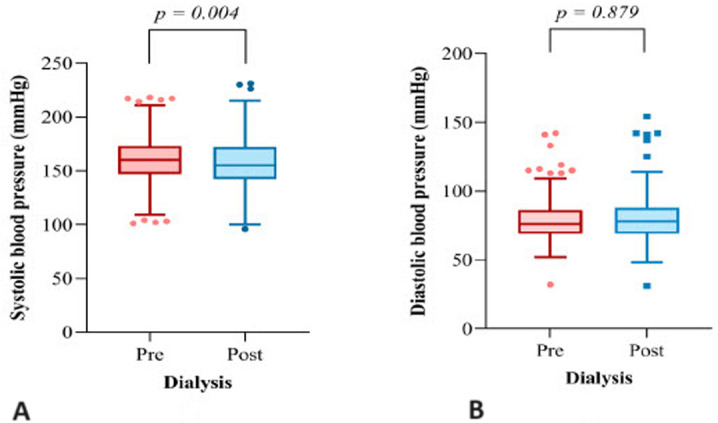
A,B) the median values and the association of pre-and post-dialysis BP (n= 314)

**Pre- and post-dialysis BP of the study participants:** the findings showed that only 16.9% (n= 53) of the 314 patients on HD had attained a controlled pre-dialysis BP target, based on the definition of controlled BP (i.e. pre-dialysis BP <140/90 mmHg). The median pre-dialysis systolic BP was 160.0 (147.0 - 173.0) mmHg, while the median post-dialysis systolic BP was 155.0 (142.0 - 172.0) mmHg. The difference in systolic BP between pre- and post-dialysis BP was statistically significant (p= 0.004). The median (IQR) pre-dialysis diastolic BP was 76 (69.0 - 86.0) mmHg, and the median post-dialysis BP was 78.0 (69.0 - 88.0) mmHg. The diastolic BP difference between pre- and post-dialysis was not statistically significant (p= 0.879) Figure 1.

**Predictors of uncontrolled pre-dialysis BP in patients on hemodialysis:** after adjusting for confounders, the multivariable model showed that participants on monotherapy had a 36% lower risk of having uncontrolled BP compared to those taking multiple medications (≥3) (aPR) 0.64: 95% confidence interval (CI), (0.45 - 0.82); p = 0.001). Participants taking BBs had a 23% lower risk of having uncontrolled BP (aPR 0.77: 95% CI (0.65 - 0.91); p = 0.002) compared to patients who were not on BBs. Moreover, participants who had hypertension for more than 120 months and those who had it for 61 to 120 months had a 24% lower probability of having uncontrolled BP than those with hypertension for more than 120 months. Participants who had dialysis less than three times per week were 20% more likely to have uncontrolled BP than those who had dialysis at least three times per week (aPR 1.20: 95% CI, (1.04 - 1.38); p = 0.011). Table 3 presents the univariate and multivariate analysis results for the predictors of uncontrolled BP among patients on dialysis.

**Table 3:** predictors of uncontrolled pre-dialysis BP among the patients on dialysis (n=314)

Variable	Univariate analysis		Multivariable analysis	
	cPR (95%CI) p-value		aPR (95% CI) p-value	
**Education status**				
Informal education	1.04 (0.71 – 1.54)	0.826	1.22 (0.84 - 1.75)	0.292
Primary education	0.70 (0.45 – 1.09)	0.118	0.75 (0.50 - 1.13)	0.167
Secondary education	1.18 (0.99 – 1.40)	0.064	1.12 (0.95 - 1.31)	0.171
Tertiary education	Ref		Ref	
**Insurance status**				
Insured	0.91 (0.83 -1.00)	0.054	1.16 (0.98 - 1.36)	0.081
Cost-sharing	Ref		Ref	
**Alcohol use**				
Current drinker	1.18 (1.12 -1.24)	<0.001	1.21 (0.92 - 1.58)	0.174
Ex-drinker	0.83 (0.69 -1.01)	0.057	0.98 (0.82 - 1.17)	0.841
Non-drinker	Ref		Ref	
**History of co morbidity**				
Yes	0.91 (0.82 -1.01)	0.088	0.95 (0.86 - 1.05)	0.294
No	Ref		Ref	
**Months since hypertension diagnosis**				
≤ 12	0.88 (0.75 -1.03)	0.103	0.84 (0.71 - 0.99)	**0.038**
13 - 60	0.96 (0.85 -1.09)	0.549	0.89 (0.79 - 1.01)	0.065
61 - 120	0.80 (0.67 -0.95)	0.013	0.76 (0.64 - 0.91)	**0.002**
>120	Ref		Ref	
**Dialysis sessions in a week**				
<3	1.18 (1.08 – 1.29)	<0.001	1.20 (1.04 - 1.38)	**0.011**
3	Ref		Ref	
**Types of medications**				
**ARBs**				
Yes	1.21 (1.15 – 1.28)	<0.001	1.01 (0.86 - 1.20)	0.885
No	Ref		Ref	
**BBs**				
Yes	0.90 (0.80 -1.00)	0.062	0.77 (0.65 - 0.91)	**0.002**
No	Ref		Ref	
**Diuretics**				
Yes	1.07 (0.97 -1.19)	0.185	0.92 (0.79 - 1.09)	0.336
No	Ref		Ref	
**CCBs**				
Yes	1.30 (1.11 -1.52)	0.001	1.04 (0.88 - 1.22)	0.685
No	Ref		Ref	
**Vasodilators**				
Yes	1.18 (1.06 -1.31)	0.003	0.94 (0.81 - 1.09)	0.420
No	Ref		Ref	
**Number of medications**				
1	0.76 (0.60 - 0.95)	**0.018**	0.64 (0.45 - 0.82)	**0.001**
2	1.07 (0.87 - 1.26)	0.597	0.89 (0.77 - 1.04)	0.156
≥3	Ref		Ref	

## Discussion

This study assessed BP control and antihypertensive medications prescribing pattern among patients with hypertension on HD in Tanzania. The findings showed that the magnitude of pre-dialysis BP control is low. The CCBs were the most prescribed antihypertensive medications. The use of CCBs, monotherapy, the number of dialysis sessions, and the duration of hypertension diagnosis were determinants of BP control among patients with hypertension on HD. The proportion of patients with hypertension on HD who attained controlled pre-dialytic BP in this study is slightly low (16.9%, n = 53) compared to what was reported previously in the United States (30%, n= 659), Pakistan (28.7%, n= 68) and Malaysia (28.9%, n= 42) [3,21,22]. Differences in the thresholds used to define BP control, as noted by Workgroup KD *et al*. 2003 who defined hypertension as pre-dialysis systolic BP >150 mmHg or diastolic BP >85 mmHg [23], or the use of antihypertensive medications, could account for the discrepancies in the results of these studies [3]. However, differences in ethnicity of the study population could also account for the difference observed. Nevertheless, the findings signify that control of BP among patients with hypertension on HD is of concern for both developed and developing countries. Our study demonstrated that CCBS, BBs, loop diuretics, and ARBs are the most prescribed antihypertensive medications, while ACEIs are the least used. Several studies reported that CCBs are significantly prescribed because of their efficiency in reducing BP even in a volume overload state and have the advantage of once-daily dosing [8,24,25]. In USA and Japan, ACEIs and ARBs are reported to be widely used as first-line antihypertensives due to their safety and efficacy in controlling cardiovascular events among chronic kidney disease patients and in the general population [12,26-28]. The observed infrequent use of ACEIs could be associated with their risk of inducing hyperkalemia caused by blockage of the renin-angiotensin-aldosterone pathway [29,30].

In this study, 24.9 % (n= 78) of our patients were using diuretics as one of their antihypertensive medications. The findings do not align with several studies that reported that diuretics are ineffective for BP control in patients with ESRD [11-13,31]. However, studies by Lemes *et al*. 2011, and Flythe *et al*. 2020, support the use of diuretics in patients on HD [32,33]. The use of diuretics in this population may be justified by the fact that they are helpful to patients on dialysis with residual diuresis to enhance urine output and prevent fluid overload [3,2]. In addition, loop diuretics in patients with residual renal function are associated with lower interdialytic weight gain and potassium levels [3,4]. Lastly, this study observed that patients with hypertension on HD using BBs, CCBs, and antihypertensive monotherapy, and those attending at least 3 dialysis sessions per week are more likely to have adequate BP control. Moreover, recently diagnosed patients with hypertension on HD can also attain adequate BP control. Several studies have also reported similar factors contributing to adequate pre-dialysis BP control in this population [25,35-40]. Other studies reported that using BBs is associated with better hypertension control and reduced cardiovascular events and death incidence in HD patients [14,15]. Specifically, carvedilol has been useful in patients with intradialytic hypertension due to its ability to improve endothelium-dependent flow-mediated vasodilatation [4,1]. The use of monotherapy is favored because of minimal drug-drug interaction and good adherence [42-44]. Our study should be interpreted in light of some limitations. The selection of stable patients may have excluded those with severe CVDs, thus overestimating the prevalence and treatment while underestimating the control of BP. Lower BP values in persons with HD are related to cardiovascular illnesses. As a result, treatment outcomes might be even better. Additionally, the non-pharmacological management of the participants, such as salt and fluid restriction, was not evaluated in our investigation, which could have improved the value of our findings.

## Conclusion

The study found that most patients with hypertension on HD have poor pre-dialysis BP control. Several antihypertensive medications are used to manage patients with hypertension undergoing HD; however, CCBs are the most prescribed. The number of dialysis sessions per week and the duration since diagnosis with hypertension were associated with uncontrolled pressure. We recommend further studies be conducted in both public and privately owned health facilities to be able to design proper interventions that will be suitable for our population.

### What is known about this topic

- *Blood pressure is poorly controlled among patients with hypertension undergoing hemodialysis;*
- *Different antihypertensive medications have been reported to work best in different populations*.

### What this study adds

- *This is among a few studies conducted on the African population known to have a diverse response to therapy, co-morbidities, and poor healthcare system, significantly affecting the management of patients with hypertension undergoing hemodialysis;*
- *This study shows a very small proportion of patients with adequate BP control compared to studies conducted in the middle- or high-income countries;*
- *We have also been able to describe the common antihypertensive medications used in our settings; determinants of inadequate blood pressure control have also been described, which provide baseline information on how we can improve the management of these patients*.
